# The Effect of Scratch-Induced Microscale Surface Roughness on Signal Transmission in Radio Frequency Coaxial Connectors

**DOI:** 10.3390/mi16080837

**Published:** 2025-07-22

**Authors:** Yuqi Zhou, Tianmeng Zhang, Gang Xie, Jinchun Gao

**Affiliations:** 1 Beijing Key Laboratory of Work Safety Intelligent Monitoring, Beijing University of Posts and Telecommunications (BUPT), Beijing 100876, China; 2 School of Information and Communication Engineering, Beijing University of Posts and Telecommunications (BUPT), Beijing 100876, China

**Keywords:** microscale scratches, RF coaxial connector, surface roughness, contact impedance, equivalent circuit modeling, signal integrity, miniaturized interconnects, microsystems reliability

## Abstract

Electrical connectors play a vital role in ensuring reliable signal transmission in high-frequency microsystems. This study explores the impact of microscale scratch-induced surface roughness on the alternating current (AC) contact impedance of RF coaxial connectors. Unlike traditional approaches that assume idealized surface conditions, controlled micro-defects were introduced at the central contact interface to establish a quantitative relationship between surface morphology and signal degradation. An equivalent circuit model was constructed to account for local impedance variations and the cumulative effects of cascaded connector interfaces. The model was validated using S-parameter measurements obtained from vector network analyzer (VNA) testing, showing strong agreement with simulation results. Experimental results reveal that the low-roughness (0.4 μm) contact surfaces lead to degraded signal integrity due to insufficient micro-contact formation. In contrast, scratch-induced moderate roughness (0.8–4.8 μm) improves transmission performance, although signal quality declines as roughness increases within this range. These effects are further amplified in multi-connector configurations due to accumulated impedance mismatches. This work provides new insight into the coupling between microscale surface features and frequency-domain transmission characteristics, offering practical guidance for surface engineering, contact design, and the development of miniaturized, high-reliability radio frequency interconnects for next-generation communication systems.

## 1. Introduction

RF coaxial connectors are critical components in miniaturized high-frequency communication modules and microsystems, where compact integration, signal fidelity, and long-term reliability are of paramount importance. In particular, the central contact interface—defined by its small-scale geometry and high current density—plays a decisive role in ensuring consistent electrical performance. However, during prolonged use in dense or dynamic microsystem environments, this interface is prone to surface degradation caused by mechanical wear, frequent mating cycles, or accidental contact damage. These processes often introduce microscale scratches and topographical defects, which can significantly alter local contact impedance and change frequency-domain transmission characteristics, especially at microwave and millimeter-wave frequencies.

In practical microsystem applications, RF connectors are seldom used in isolation; instead, they are commonly integrated into cascaded transmission networks comprising multiple connectors and tightly matched transmission lines. While the line impedance is generally well controlled through precision fabrication, the degraded contact quality of individual connectors can become a key bottleneck in overall system performance. Therefore, a quantitative understanding of how microscale surface damage affects the high-frequency behavior of contact interfaces is essential for the design and optimization of robust interconnect structures in compact RF systems.

Previous studies have extensively investigated methods for analyzing RF connectors by establishing equivalent circuit models. Paleček et al. [1] proposed two techniques for parameter extraction of RF connectors: one based on S-parameter measurements and the other combining mathematical modeling with a differential evolution algorithm to estimate parasitic element values. Mandic et al. [2] developed a broadband equivalent circuit model for RF connectors that incorporates multiple coupling networks. Jin et al. [3] developed an equivalent circuit model to investigate the frequency and power dependence of passive intermodulation (PIM) in RF connectors, primarily caused by contact surface nonlinearity in conjunction with the nonlinear properties of ferromagnetic materials.

In addition to these modeling approaches, research has also addressed the electrical degradation of RF connectors under various aging and failure scenarios. For instance, Yang et al. [4] proposed a time-domain reflectometry (TDR) method to predict passive intermodulation distortion caused by loose coaxial connectors. Lin et al. [5] reported the optimization of pin geometry for improved impedance matching. Furthermore, the impact of connector geometry, mechanical tolerances, and the condition of surrounding conductors on reflection characteristics has been examined by Mubarak et al. [6] and Wang et al. [7].

At the interface scale, the influence of surface roughness and contact topography has gained increasing attention due to its strong correlation with contact impedance. Zhang et al. [8] developed a roughness-based contact impedance model validated through field simulations and experiments. Fractal-based models by Lei et al. [9] and Capelli et al. [10] further enabled geometric characterization of surface morphology. Additional studies by Rachman et al. [11], Angadi et al. [12], Wilson et al. [13], and Malucci [14] have demonstrated how surface microgeometry, contact pressure, and plating material affect electrical and thermal resistance, using both experimental and multi-scale theoretical approaches.

Furthermore, recent research has shown that both machining profile geometry and data-driven modeling play important roles in surface roughness control and prediction: Alinaghizadeh et al. [15] revealed how curvature and pass number, during wire EDM, influence recast layer uniformity, while Yang et al. [16] introduced a hybrid virtual sample generation model to predict surface roughness in data-limited polishing processes.

While these efforts have provided critical insights into interface-level phenomena, limited work has focused on the quantitative impact of scratch-induced roughness on frequency-dependent performance metrics, such as S-parameters. Moreover, existing modeling frameworks often lack the integration of physically measured surface characteristics with system-level simulations and experiments.

In this study, a systematic approach is developed to quantitatively evaluate how microscale scratches affect the high-frequency electrical behavior of RF coaxial connectors. A series of 2.92 mm connector samples with intentionally varied scratch morphologies were fabricated, and their contact interfaces were characterized using high-resolution surface profilometry. Based on the extracted morphological parameters, alternating current (AC) contact impedance was analytically modeled, incorporating both the skin effect and asperity-level interactions. An equivalent circuit model was then constructed to reflect localized impedance variations and their cumulative influence in cascaded connector systems.

The simulated S-parameters derived from the model were validated against experimental measurements, showing strong consistency across a wide frequency range. The results reveal that low-roughness contact (0.4 μm) leads to degraded signal transmission due to insufficient micro-contact formation, while moderate roughness (0.8–4.8 μm) improves performance; however, a decline in signal integrity is observed as roughness increases within this range, and this degradation is amplified in multi-connector systems. This work establishes a unified, quantitative framework for linking contact surface microgeometry to frequency-domain transmission characteristics, offering valuable insights for the design, fabrication, and reliability assessment of miniaturized RF interconnects in high-frequency communication platforms.

## 2. Sample Preparation and Data Collection

### 2.1. Connector Sample Fabrication

**Figure 1:**
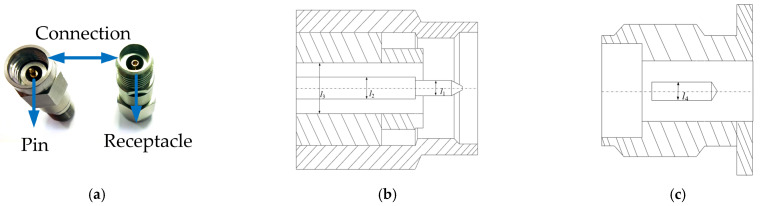
Mechanical structure of the Precision 2.92 mm RF Connector: (**a**) external view; (**b**) dimensional specifications of the pin (*l*_1_ = 0.93 mm, *l*_2_ = 1.27 mm, *l*_3_ = 2.93 mm); and (**c**) dimensional specifications of the receptacle (*l*_4_ = 1.27 mm).

In this study, Precision 2.92 mm RF connectors were selected as experimental samples due to their widespread application in high-frequency communication systems and their stringent design requirements for signal integrity, as shown in Figure 1a. Structurally, these connectors consist of a central conductor, a dielectric insulator, and an outer conductor. To ensure a consistent 50 Ω characteristic impedance throughout the transmission path, the dimensions of these three elements—specifically the ratio between the inner and outer conductor diameters—must be precisely matched during design and fabrication. The detailed dimensional specifications of the Precision 2.92 mm RF connector are shown in Figure 1b,c.

**Figure 2:**
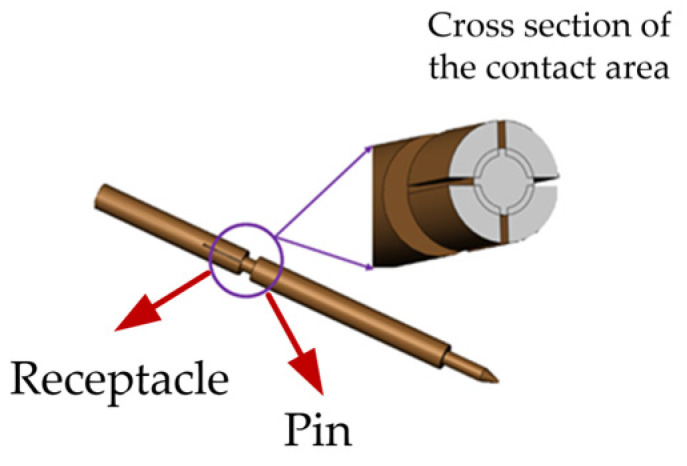
Schematic of the contact interface between the pin and receptacle of the central conductor in the precision 2.92 mm RF connector with a non-fixed elastic connection.

In addition to dimensional matching, the connectors require carefully engineered interconnect strategies. The outer conductors are joined via a robust, threaded coupling mechanism, which ensures mechanical and electrical stability. In contrast, the central conductors rely on a non-fixed, spring-loaded pin-and-receptacle configuration. This configuration forms an electrical path through elastic contact, which is inherently less stable and highly sensitive to both mechanical and surface conditions. In particular, the contact region at the center conductor is subject to micro-scale asperity contact, where local resistance and current distribution are strongly influenced by the surface roughness and plating quality of the contact interface. Figure 2 depicts the contact structure between the pin and receptacle of the central conductor in the precision 2.92 mm RF connector, emphasizing the non-fixed, elastic interface at the central conductor contact.

To emulate realistic variations caused by wear and aging, this study adopts a systematic method for fabricating connector samples with precisely controlled scratch depths at the central contact interface. All samples were initially machined from brass substrates and then subjected to a two-step electroplating process: a 2.6 μm nickel layer was first deposited to improve adhesion and provide diffusion resistance, followed by a 2.62 μm electroplated hard gold layer to ensure excellent electrical conductivity and corrosion resistance.

The maximum surface height (S_z_) is one of the commonly used three-dimensional surface roughness parameters. It represents the vertical distance between the highest peak (Z_max_) and the lowest valley (Z_min_) within the measured region:

(1) $$S_{Z}=Z_{\max}-Z_{\min}$$

Due to its definition, the maximum surface height is particularly suitable as a representative parameter for characterizing scratch-induced surface roughness variations. When the scratch size and distribution are relatively uniform, the maximum surface height provides valuable insight into the degree of surface degradation at the contact interface. As an indicator of extreme topographical variations, it is especially relevant in assessing how localized protrusions or deep scratches affect the electrical contact performance.

**Figure 3:**
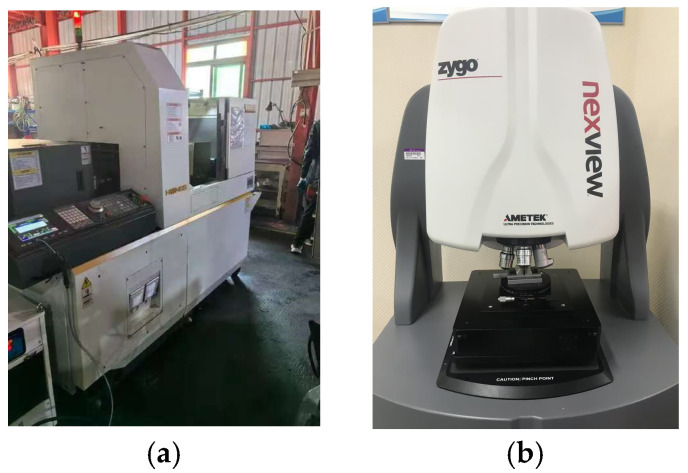
Preparation and measurement of scratch-induced surface roughness on the precision 2.92 mm RF coaxial connector: (**a**) precision CNC lathe machining; and (**b**) ZYGO 3D white light interferometric surface profilometry.

The maximum surface height was selectively modulated by introducing controlled turning-induced tool marks using precision CNC lathe machining, as shown in Figure 3a. The process specifically targeted the central contact region of the connector pin while preserving its overall geometric and dimensional integrity. A cemented carbide tool with a nose radius of 1 mm was utilized under dry cutting conditions to avoid surface contamination. By systematically adjusting key turning parameters—such as feed rate, spindle speed, and depth of cut—three distinct levels of surface roughness (S_z_ = 0.8 μm, 3.2 μm, and 4.8 μm) were achieved to simulate varying degrees of scratch-induced damage. In addition, a reference sample with a surface roughness of S_z_ = 0.4 μm was retained in the as-machined state, without any intentional scratch introduction. This surface condition corresponds to that of a standard commercial connector and is considered representative of a typical, unscratched interface for comparison purposes.

The resulting turning marks produced periodic surface textures that simulate typical wear-induced features encountered in repeated mating and unmating cycles. As a representative parameter of extreme surface topography, S_z_ effectively captures both the intrinsic roughness and scratch-equivalent depth introduced by the machining process. This controlled fabrication approach enables reproducible and isolated investigation of contact surface morphology, without altering the nominal connector geometry or its characteristic impedance. The methodology thus provides a practical and application-relevant platform for evaluating the impact of machining-induced surface degradation on high-frequency signal transmission behavior.

**Figure 4:**
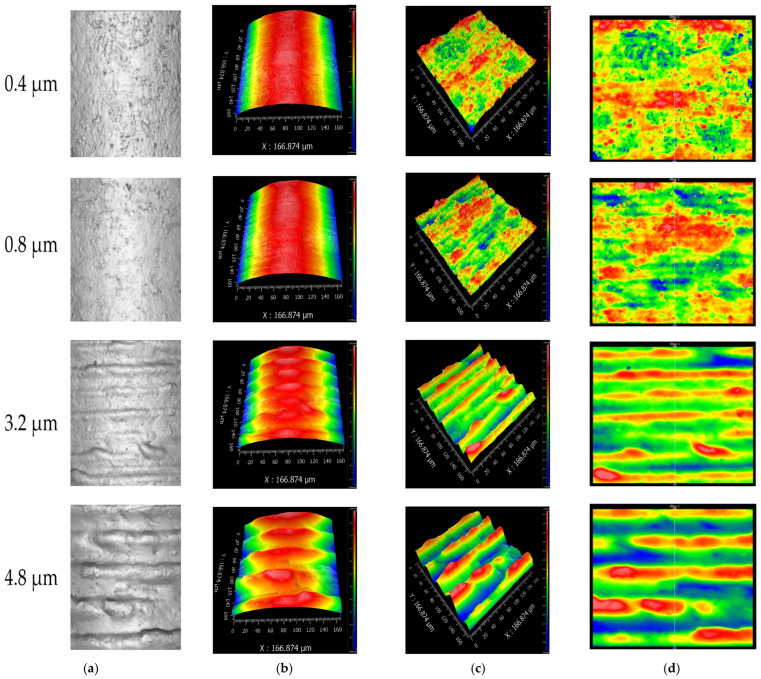
Microscopic surface morphology of the connector: (**a**) actual surface topography of the pin; (**b**) modeled microscale surface profile; (**c**) flattened surface morphology after removing curvature effects; and (**d**) frontal view of the contact surface microstructure.

The surface topography of each contact interface was subsequently measured using a ZYGO three-dimensional white light interferometric surface profilometer, as shown in Figure 3b. The measurement and processing procedures for the surface morphology are illustrated in Figure 4a. For each sample, a region of 166.874 μm × 166.874 μm—the maximum area measurable in a single scan by the instrument—was selected on the central contact area of the pin for topographical analysis. To ensure statistical reliability and spatial consistency, three equally spaced regions were measured on each contact surface to extract surface parameters. Figure 4a shows the measured surface topographies of the pin from connectors with different maximum surface heights (S_z_). As the maximum height of the contact surface increases—precisely adjusted via machining parameters—the simulated insertion wear marks become more prominent, with the depth of scratches exhibiting systematic and repeatable variation. This results in a visibly rougher surface morphology that reflects different levels of mechanical degradation. Figure 4b presents the intermediate data processing step, where the raw topography is modeled to facilitate mathematical treatment and eliminate the influence of the pin’s curvature radius. Figure 4c displays the resulting microstructure of the contact surface after curvature compensation, revealing the true contact interface. Figure 4d provides a frontal view of the processed contact surface morphology, suitable for micro-contact analysis. Based on the above measurement results, the scratches in this study were found to be relatively uniformly distributed, with minimal variation in the peak heights of individual asperities and similarly small differences in valley depths across the contact surface. Therefore, the maximum surface height (S_z_) was selected as a representative roughness parameter to characterize the scratch-induced condition of the contact interface.

Based on the microscopic morphology shown in Figure 4, key surface parameters under varying maximum surface height conditions were statistically extracted and summarized in Table 1. In addition to the maximum surface height, the average asperity spacing (*b*), average asperity width (*a*), and average asperity valley distance (*a*_1_) were obtained from the measured surface profiles. Average asperity width was defined as the lateral extent of surface protrusions above the mean surface height, while spacing and valley distance were derived from adjacent peak intervals. Asperity density was estimated by counting the number of features within a fixed area.

**Figure 5:**
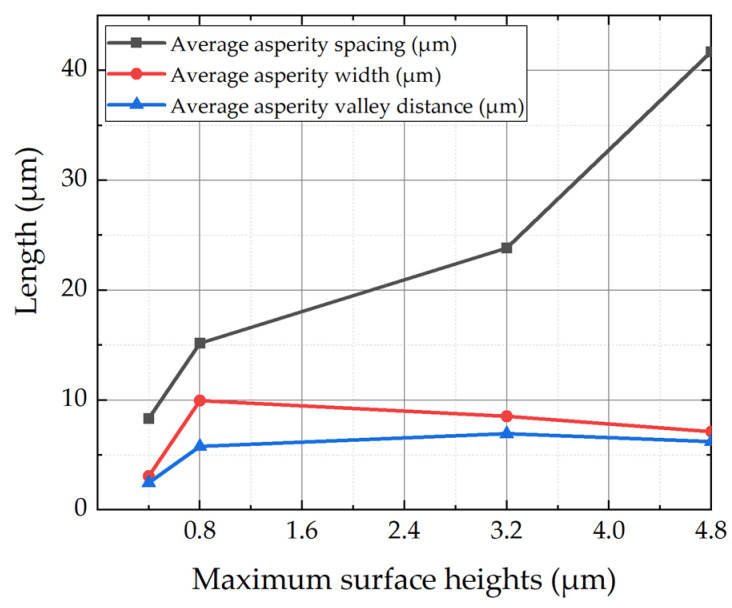
Variation of surface morphology parameters with maximum surface height.

As shown in Figure 5, average asperity spacing increases monotonically with S_z_. In contrast, both asperity width and valley distance show non-monotonic trends: increasing initially and then slightly decreasing. The peak asperity width appears at S_z_ = 0.8 μm, while the valley distance reaches a maximum at S_z_ = 3.2 μm.

This behavior reflects the mechanics of scratch formation using a conical drill bit with controlled penetration depth. Shallow scratches cause minimal material displacement and small ridges. At intermediate depths, plastic flow becomes significant, enlarging asperities and valleys. At greater depths, lateral flow is constrained and material is displaced downward. In addition, deeper scratches penetrate the hard gold layer (153 HV, 1.5 × 10^9^ Pa) and reach the softer nickel substrate, whose elastic recovery upon unloading further reduces asperity width and valley distance.

The number of asperities was determined from surface topography data acquired using a ZYGO three-dimensional white light interferometric surface profilometer. In each measured region, surface features exceeding the mean height were identified as asperities. The asperity count in each region was obtained, and the average across the three regions was calculated as the final asperity number (*N*) corresponding to the given maximum surface height.

Despite variations in surface morphology, SEM measurements show minimal changes in apparent contact area (=0.167 mm^2^), indicating that contact area is more strongly influenced by mechanical alignment between the pin and receptacle. These surface parameters serve as essential inputs for the high-frequency equivalent circuit model and subsequent signal integrity analysis.

### 2.2. Evaluation of Pin–Receptacle Contact Force

While surface roughness—particularly scratch depth and average asperity width—has been demonstrated to significantly influence high-frequency signal transmission, it is not the sole factor determining contact performance. The mechanical contact force at the pin–receptacle interface also plays a critical role by governing the real contact area and stabilizing the electrical conduction paths.

To quantitatively assess the stability of electrical conduction, the mechanical force between the pin and receptacle was experimentally measured and calculated under controlled assembly conditions. These measurements enabled correlation analyses between the mechanical force and the resulting S-parameter behavior under varying surface conditions. The results indicate that contact force and surface morphology are coupled factors, both contributing to the overall impedance characteristics and signal integrity of the connector system. Therefore, accurate prediction and optimization of connector performance require consideration of both surface topography and interfacial loading.

**Figure 6:**
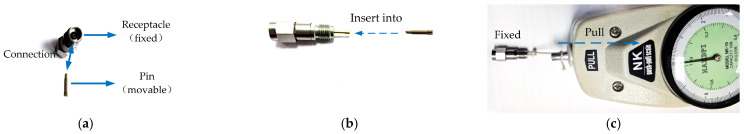
Measurement procedure for maximum static friction force: (**a**) fixed receptacle of the connector and movable pin; (**b**) the interconnection between the fixed receptacle and the movable pin; and (**c**) friction force measurement during pin extraction.

Since the central and outer conductors were manufactured separately and assembled in a subsequent process, multiple standalone central conductors were fabricated for each surface condition, in addition to those used for signal integrity testing, as shown in Figure 6a. These individual central conductor pins were mated with the corresponding receptacles, and the removal force (maximum static friction force) was measured using a dial-type push–pull gauge (Figure 6b,c). The mechanical contact force was subsequently calculated based on the measured maximum static friction force and the coefficient of friction:

(2) $$F_{n}=\frac{f_{\max}}{\mu }$$

where *f*_max_ is the maximum static friction force, *F_n_* is the contact force, and *μ* is the friction coefficient. The value of *μ* can be measured using a reciprocating friction and wear testing machine (tribometer). The frictional characteristics and corresponding contact forces for contact surfaces with different scratch profiles are summarized in Table 2. A comprehensive understanding of the connector contact structure was achieved through detailed measurement and statistical analysis of the contact surface topography, combined with the calculation of mechanical contact force.

## 3. Mathematical Modeling

### 3.1. Modeling of High-Frequency Contact Resistance

**Figure 7:**
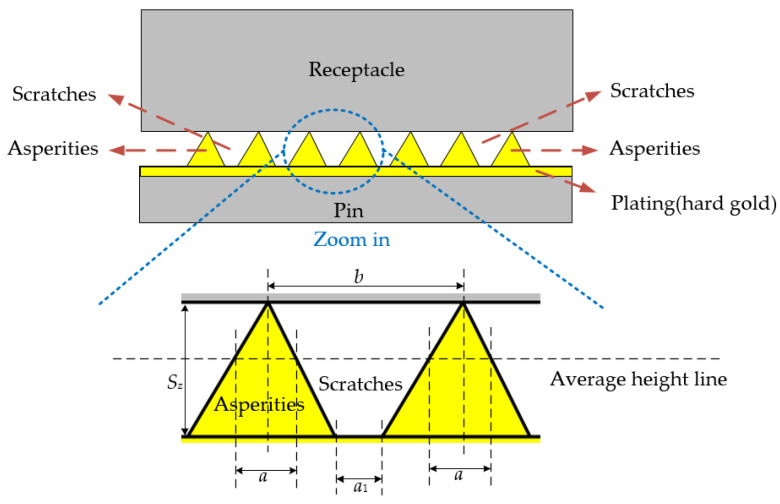
Schematic illustration of asperity contacts between the pin and receptacle.

According to Holm’s theory [17], electrical contact between conductive surfaces is typically considered to occur at microscopic asperities. Based on microscopic observations of the pin surface of a precision 2.92 mm RF connector, we found that actual contact occurs primarily between the receptacle and the elevated regions surrounding the scratches on the pin, which are theareas that protrude significantly above the average surface height. Under DC conditions, the contact resistance is mainly caused by current constriction near the scratches, where the current flow becomes concentrated. A schematic illustration of the contact between pin asperities and the receptacle is shown in Figure 7, which also provides a magnified view of the asperity morphology. According to electrical contact theory, the DC contact resistance can be expressed as follows [18]:

(3) $$R_{c\text{\_}\mathrm{DC}}=\sqrt{\frac{\rho^{2}\pi DH}{4F_{n}}},$$

where *ρ* is the electrical resistivity of the electroplated hard gold layer, *H* is the Vickers hardness, and *D* is an empirical coefficient of order unity. Scratches are relatively uniformly distributed within the contact area; therefore, the asperities formed around these scratches also establish fairly uniform contact with the receptacle.

The asperity density (η) was calculated by averaging the number of asperities observed within each measured region and normalizing by the corresponding area. Specifically, for each maximum surface height, the number of asperities was statistically determined across multiple 166.874 μm × 166.874 μm surface regions, and the average value was used to compute *η*.

(4) N=η⋅S

where *N* is the average number of asperities and *S* is the area of the surface region used for roughness measurement with the ZYGO three-dimensional white light interferometric surface profilometer. The resulting asperity densities for different maximum surface height values are summarized in Table 2.

Unlike direct current (DC) conditions, high-frequency signal transmission induces the skin effect, whereby the current is confined to the near-surface region of the center conductor in the precision 2.92 mm RF connector. The corresponding skin depth of the center conductor can be calculated using the following expression:

(5) $$\delta =\sqrt{\frac{\rho }{\pi f\mu_{0}\mu_{r}}},$$

where *f* is the signal frequency, *μ*_r_ is the relative permeability of the conductor, *μ*_0_ is the magnetic permeability of free space, and *ρ* is the resistivity of the conductor. Based on the skin depth and current constriction effects, the high-frequency AC resistance can be calculated as described in [2]:

(6) $$R_{c\text{\_}\mathrm{AC}}=\frac{\rho }{\pi \delta }\ln\left(\frac{r_{\mathrm{cylinder}}}{g}\right)+\frac{0.14\rho \delta^{0.2}}{a^{1.24}}-0.8\times 10^{-3},$$

where *a* is average asperities width, *r*_cylinder_ = 100 μm is the dimension of the region of the contact point, and $g=\rho /2R_{c\text{\_}\mathrm{DC}}$ is the constriction radius of plating.

**Figure 8:**
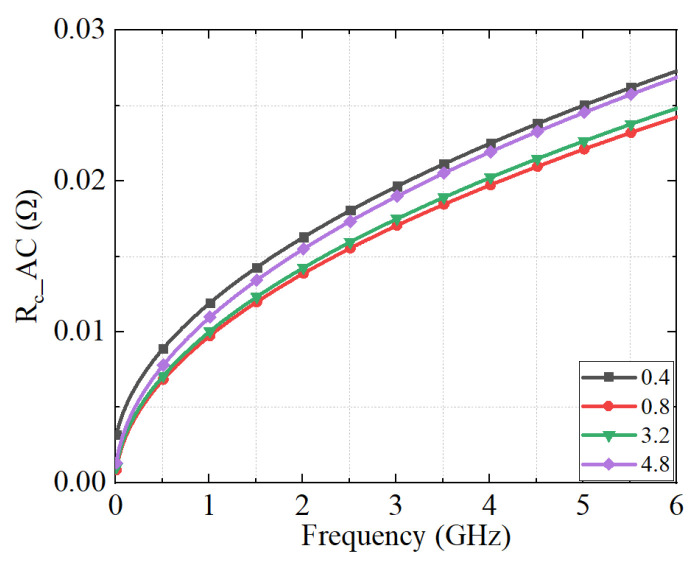
The contact resistance of connectors with different S_z_ at different frequencies.

The relationships between the AC contact resistance the of connectors with different roughness and frequency are shown in Figure 8.

### 3.2. Modeling of High-Frequency Contact Impedance

From a microscopic perspective, the surface of the pin in a precision 2.92 mm RF connector exhibits not only micro-asperities but also a substantial number of grooves formed due to scratches. These grooves introduce localized air gaps, which act as insulating media owing to the reduced physical contact between the mating surfaces. When the center conductors are mated, a vertical separation exists between the recessed regions of the pin surface and the corresponding receptacle interface. This configuration leads to parasitic capacitive coupling across the contact interface. Compared to micro-asperities, the volumetric and geometric characteristics of scratch-induced gaps contribute to a significantly stronger capacitive effect. Accordingly, this study focuses specifically on the capacitance arising from scratch-induced features.

**Figure 9:**
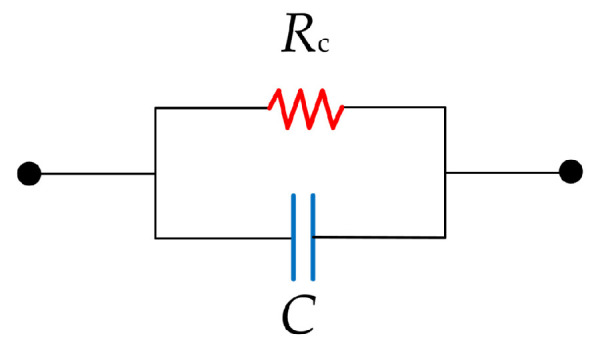
The RC impedance network diagram of a single contact point.

Under high-frequency operation (in the GHz range), time-varying electric fields induce displacement currents across these parasitic capacitances, facilitating electromagnetic energy transfer even in the absence of full mechanical contact. Consequently, the overall impedance of the interconnect is governed not only by contact resistance but also by capacitive coupling effects, as illustrated in Figure 9. This parasitic capacitive path operates in parallel with the conventional ohmic contact, forming a composite transmission mechanism that may impact high-frequency signal integrity.

**Figure 10:**
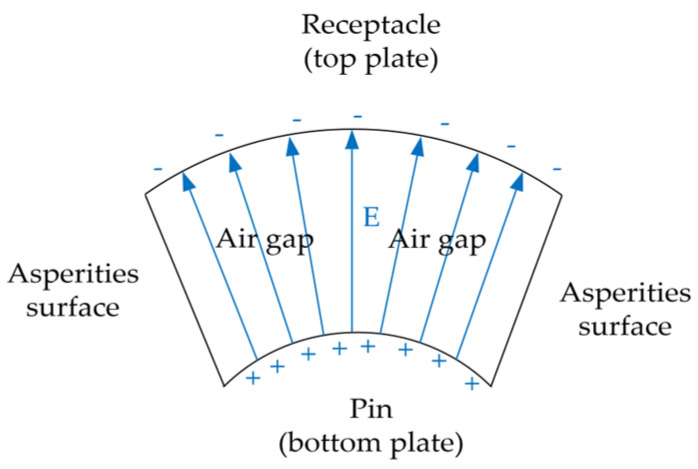
Cross-sectional schematic of a ring-shaped parallel-plate capacitor formed by the pin, scratch-induced air gap, and receptacle.

As observed from the surface profilometry images, the designed samples exhibit relatively uniform scratch distributions, with minimal variation in scratch depths. Based on the scratch morphology, the air gaps can be simplified into a V-shaped structure. Each pin–scratch gap–receptacle configuration can thus be modeled as an annular parallel-plate capacitor, as illustrated in Figure 10. The annular capacitor structure is analogous to an unwrapped strip-type capacitor; therefore, its capacitance can be approximated using the average area method:

(7) $$C_{n}=\varepsilon \varepsilon_{r}\frac{A_{b}+A_{t}}{2d}$$

where *C*_n_ denotes the capacitance contributed by a single scratch-induced gap, *A*_b_ and *A*_t_ represent the areas of the bottom and top electrodes, respectively, and *d* is the separation distance between the electrodes, corresponding to the average scratch depth (*S*_z_).

The bottom electrode area *A*_b_ can be estimated using the pin diameter and the average asperity valley distance:

(8) $$A_{b}=\pi l_{1}a_{1}$$

The top electrode area *A*_t_ can be estimated by considering the pin diameter and the average asperity spacing:

(9) $$A_{t}=\pi \left(l_{1}+d\right)b$$

According to Equation (4), the number of asperities within the apparent contact area can be estimated by multiplying the asperity density *D* with the apparent contact area *A*. Elevated asperities—typically originating from surface scratches—tend to form localized separations from the opposing contact surface, thereby generating air gaps. Assuming a relatively uniform distribution of scratch-induced asperities, a one-to-one correspondence between elevated asperities and capacitive gaps is considered. Accordingly, the number of capacitive gaps *N* is taken to be approximately equal to the number of asperities that are sufficiently elevated to prevent direct mechanical contact with the mating surface.

As the air gaps between the pin and receptacle are spatially isolated, the associated parasitic capacitances can be regarded as electrically connected in parallel. The total capacitance can thus be expressed as follows:

(10) $$C=\varepsilon \varepsilon_{r}\frac{\pi l_{1}a_{1}+\pi \left(l_{1}+d\right)b}{2d}\cdot A_{a}\eta$$

where *C* represents the total parasitic capacitance of the connector and *A*_a_ denotes the apparent contact area. The *R*_C_ impedance network of the conductor contact region within the connector is modeled as follows:

(11) $$\frac{1}{Z}=\frac{1}{\frac{\rho }{\pi \delta }\ln\left(\frac{r_{\mathrm{cylinder}}}{g}\right)+\frac{0.14\rho \delta^{0.2}}{a^{1.24}}-0.8\times 10^{-3}}+\varepsilon \varepsilon_{r}\pi fA_{a}\eta \cdot \frac{\pi l_{1}a_{1}+\pi \left(l_{1}+d\right)b}{d}j$$

Based on the above equations, the contact resistance and capacitance between the pin and receptacle were calculated for different scratch depths and surface roughness levels. These values were subsequently used to derive the equivalent circuit model, providing input parameters for the following circuit simulation analysis.

## 4. Equivalent Circuit Analysis and Simulation

### 4.1. Equivalent Circuit Model Development

**Figure 11:**
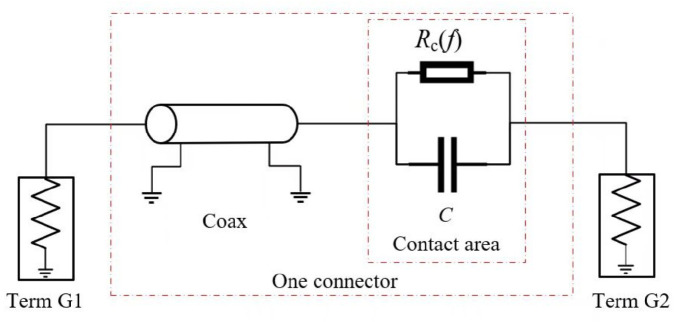
The equivalent circuit model of one connector.

To quantitatively assess the influence of surface roughness and cascading effects on the high-frequency performance of the connector system, an equivalent circuit model was established, as illustrated in Figure 11. This model serves to bridge the physical contact phenomena and the electrical signal behavior, enabling detailed analysis of signal degradation mechanisms induced by contact imperfections and multi-connector assemblies.

The equivalent circuit model consists of four main components: Term G1, a coaxial transmission line segment, a contact interface, and Term G2, all implemented within circuit simulation software. Term G1 and Term G2 represent the external signal input and output ports of the connector, respectively.

For cascaded connector configurations, the model is expanded by serially combining multiple coaxial transmission line segments and discrete contact interfaces. Each contact interface is parameterized by frequency-dependent contact resistance *R*_c_ (*f*) and parasitic capacitance *C*, which collectively capture the complex impedance variations introduced by surface roughness and micro-contact phenomena.

Due to the cumulative effects of signal reflections and attenuation at successive interfaces, cascading multiple connectors can exacerbate impedance mismatches and insertion losses, ultimately degrading overall high-frequency transmission performance. The proposed equivalent circuit framework enables a comprehensive evaluation of both localized contact interface behavior and system-level signal integrity in multi-connector configurations.

### 4.2. Results and Discussion of the Simulation

**Figure 12:**
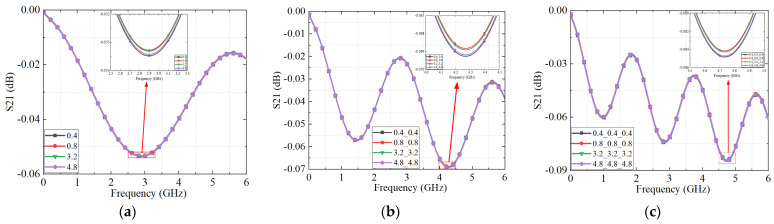
The S_21_ parameters of the connectors with different roughness obtained by an equivalent circuit model: (**a**) one connector; (**b**) two connectors with the same roughness cascade; and (**c**) three connectors with the same roughness cascade.

The S_21_ results for connectors with varying surface roughness levels, derived from the equivalent circuit model, are shown in Figure 12. As illustrated, the simulated S_21_ parameter exhibits frequency-dependent fluctuations due to periodic impedance mismatches introduced by surface irregularities across the operating bandwidth. The simulated roughness conditions correspond to the maximum surface height (S_z_) values, which were precisely controlled through machining-induced scratch depths, as detailed in earlier sections. The case with 0.4 μm surface roughness represents connectors without significant scratches, serving as a baseline for comparison.

Notably, the connector with the maximum surface height of 0.4 μm exhibits the lowest S_21_ magnitude, indicating the highest transmission loss and poorest signal integrity among all simulated cases. This seemingly counterintuitive result arises because a relatively smooth surface lacks sufficient micro-asperities to form stable micro-contact spots. As a result, the reduced real contact area limits current conduction pathways and increases contact resistance.

As the scratch-induced maximum surface height and overall roughness increase, the S_21_ performance initially improves but begins to degrade beyond a certain threshold. Within the moderate roughness range (0.8–4.8 μm), a gradual decline in S_21_ is observed with increasing the scratch-induced maximum surface height, suggesting that excessive surface scratches reduce the effective contact area and confine current flow to fewer, narrower constriction channels. Under high-frequency excitation, this intensifies current crowding, elevates constriction resistance, and ultimately impairs signal integrity.

**Figure 13:**
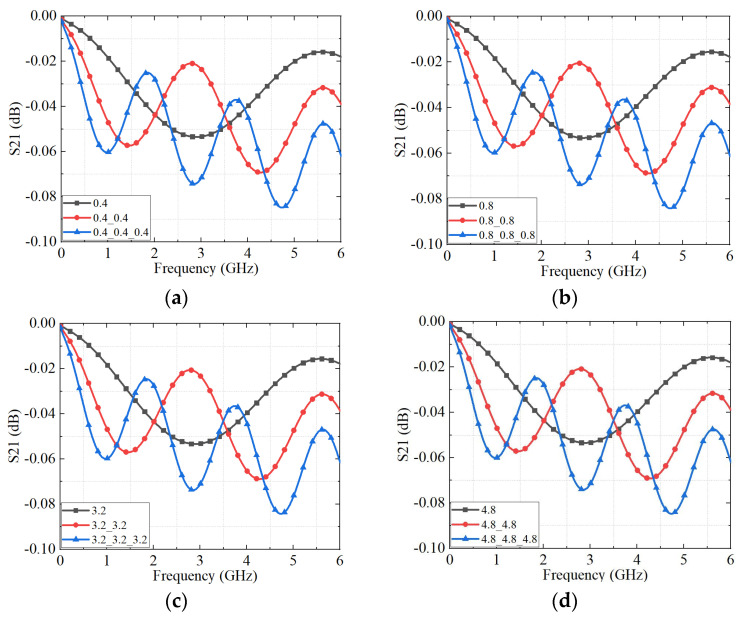
The S_21_ parameters of the connectors with different connectors cascade obtained by an equivalent circuit model. (**a**) The roughness of each connector is 0.4 μm; (**b**) the roughness of each connector is 0.8 μm; (**c**) the roughness of each connector is 3.2 μm; and (**d**) the roughness of each connector is 4.8 μm.

To further assess the impact of cascading effects, Figure 13 presents the S_21_ responses for different numbers of connected interfaces. As additional connectors are introduced, cumulative impedance discontinuities and reflection losses arise at each interface, progressively amplifying insertion loss and degrading signal transmission. Moreover, Figure 13 shows that the number of periodic oscillations in the S_21_ curve increases with the number of cascaded connectors, regardless of surface roughness. This behavior is attributed to the extended transmission path length, which results in longer effective electrical length and more pronounced standing wave patterns. The progressive signal degradation highlights the critical role of interface quality and impedance continuity in multi-connector assemblies.

By simulating the effects of surface morphology and connector cascading, the proposed model enables quantitative prediction of key high-frequency performance, including S-parameters. These simulation results provide theoretical and computational insight into how microscale surface parameters and multi-connector cascading influence signal integrity in RF interconnect systems.

## 5. Experimental Verification

### 5.1. Experimental Setup

**Figure 14:**
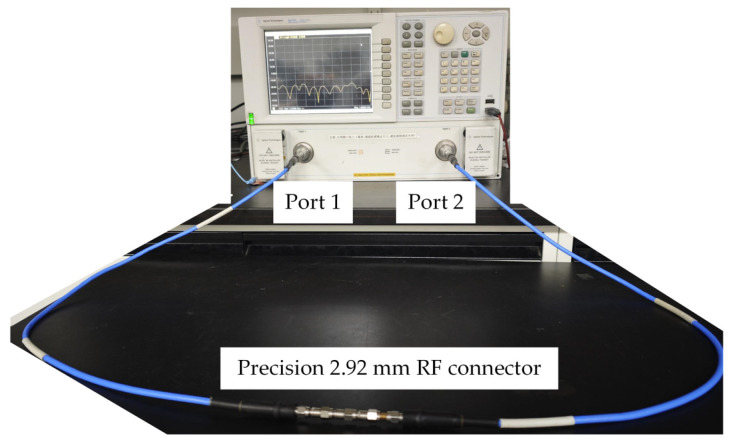
Vector network analyzer-based test system for evaluating high-frequency performance of the connectors under different surface and cascading conditions.

To validate the proposed models and the calculated electrical parameters, the previously fabricated connectors with different scratch-induced maximum surface height were used as experimental samples. For each scratch depth level, at least three connector samples were prepared to ensure repeatability. The maximum surface height values of the connectors were 0.4 μm, 0.8 μm, 3.2 μm, and 4.8 μm, respectively. In addition, three cascading configurations were established: a single connector, two connectors in cascade, and three connectors in cascade. The high-frequency measurement setup is shown in Figure 14.

The samples were connected to a vector network analyzer (VNA) through two high-frequency test cables. Port 1 and Port 2 were designated as the input and output ports, respectively. S-parameter measurements were conducted using 201 sampling points. Prior to testing, Short-Open-Load-Thru (SOLT) calibration was performed to eliminate the influence of cables and fixtures. The measurement frequency range was set from 10 MHz to 6 GHz.

### 5.2. Results and Discussion of the Measurement

**Figure 15:**
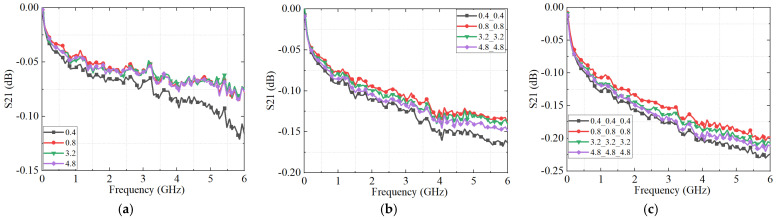
The measured S_21_ parameter of the connectors with different roughness. (**a**) one connector; (**b**) two connectors with the same roughness cascade; and (**c**) three connectors with the same roughness cascade.

**Figure 16:**
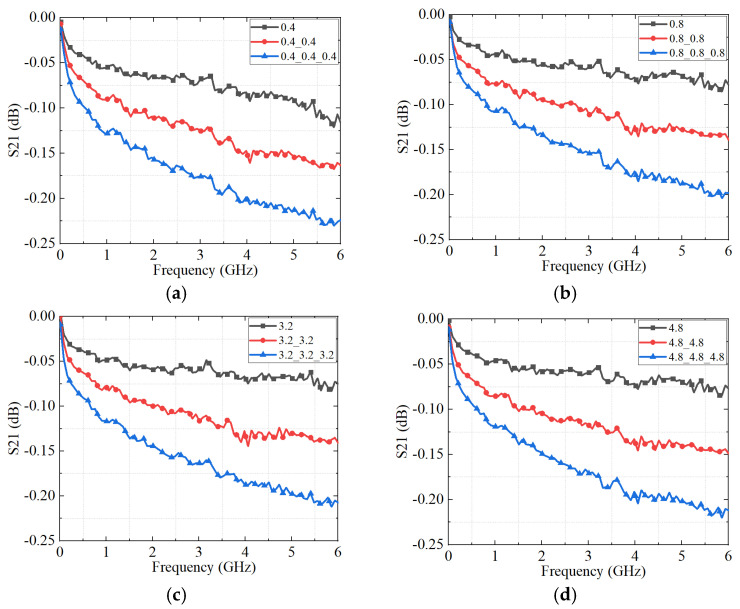
The S_21_ parameters of the connectors with different connector cascades obtained by a VNA. (**a**) The roughness of each connector is 0.4 μm; (**b**) the roughness of each connector is 0.8 μm; (**c**) the roughness of each connector is 3.2 μm; and (**d**) the roughness of each connector is 4.8 μm.

The measured S_21_ results for the connectors with different scratch-induced maximum surface height levels and varying numbers of cascaded connectors are presented in Figure 15 and Figure 16, respectively. As shown in Figure 15, the S_21_ parameter generally decreases with increasing surface roughness. However, an exception is observed at a surface roughness of 0.4 μm, where the highest transmission loss occurs. This result indicates that both excessively smooth and excessively rough contact surfaces degrade signal transmission performance. This experimental trend aligns well with the simulation predictions, reinforcing the validity of the proposed model.

Furthermore, the influence of the scratch-induced maximum surface height becomes progressively amplified as the number of cascaded connectors increases, highlighting the cumulative nature of impedance discontinuities introduced by roughness-induced contact variations. These findings emphasize the critical role of surface morphology control in maintaining signal integrity, particularly in complex interconnect systems where multiple connectors are employed.

As shown in Figure 16, the S_21_ parameter exhibits a pronounced decline with the increasing number of cascaded connectors, regardless of surface roughness. This degradation arises primarily from impedance mismatches introduced at each interface, which accumulate with the addition of more connectors and extend the overall transmission path length.

A comparison between Figure 13 and Figure 16 further demonstrates good agreement between the experimental measurements and the simulation results, thereby validating the accuracy of the proposed equivalent circuit model. For instance, in the case of the three-cascaded connector sample with a surface roughness of 3.2 μm, the simulated and measured insertion loss values at 6 GHz are −0.085 dB and −0.205 dB, respectively. The absolute deviation of 0.12 dB falls within the acceptable range for microwave and millimeter-wave applications.

The minor discrepancy between the simulation and measurement results can be attributed to several factors, including simplifications in the modeling process (such as the neglect of certain inductive effects at the electrical contact interface and conductor and dielectric losses), inevitable calibration uncertainties, fabrication tolerances, and the residual influence of the female-to-female coaxial adapters used to interface the test samples with the measurement cables.

Overall, these results not only clarify the coupling mechanisms between scratch-induced roughness, cascading effects, and high-frequency signal degradation, but also provide a basis for developing sensor-based diagnostic strategies to monitor surface degradation in real time. The proposed framework offers new insights into the quantitative evaluation and optimization of connector contact performance in high-frequency communication systems.

## 6. Conclusions

This study systematically investigated the impact of scratch-induced surface roughness on the high-frequency electrical performance of RF coaxial connectors, with emphasis on microscale contact interface behavior. By characterizing surface morphology and modeling corresponding AC contact impedance, an equivalent circuit framework was established to predict S-parameter responses under varying roughness conditions and connector cascades. The model demonstrated strong agreement with experimental measurements obtained via vector network analysis, validating its predictive accuracy.

The results highlight two competing degradation mechanisms. Overly smooth surfaces (0.4 μm) lack sufficient micro-asperities, resulting in limited contact area, elevated resistance, and poor signal transmission. Conversely, excessively rough surfaces introduce deep scratches and non-uniform contact regions, which concentrate current in narrow constriction paths and exacerbate impedance discontinuities under high-frequency excitation.

Furthermore, in cascaded configurations, roughness-induced impedance mismatches accumulate across interfaces, amplifying insertion loss and degrading overall transmission quality. This effect becomes increasingly critical in miniaturized RF systems where multiple interconnects are densely integrated.

By linking micro-scale surface morphology to S-parameter responses, this work offers a quantitative framework for evaluating and optimizing signal transmission performance in RF connectors. The findings offer practical guidance for surface engineering, interconnect design, and long-term reliability assurance in high-frequency microsystems and compact communication platforms.

## Figures and Tables

**Table 1:** Summary of microscale surface roughness features.

Maximum Surface Heights *S_Z_* (μm)	Average Asperity Spacing *b* (μm)	Average Asperity Width *a* (μm)	Average Asperity Valley Distance *a*_1_ (μm)	Number of Asperities *N*
0.4	8.33	3.07	2.47	7.00
0.8	15.17	9.94	5.78	6.50
3.2	23.84	8.52	6.94	4.67
4.8	41.72	7.12	6.21	5.34

**Table 2:** Key parameters for mechanical and contact behavior analysis.

Maximum Surface Heights *S*_Z_ (μm)	Friction Coefficient *μ*	Contact Force *F*_n_ (N)	Asperity Density *η* (×10^8^/m^2^)
0.4	0.263	4.56	2.514
0.8	0.289	6.92	2.334
3.2	0.379	4.35	1.677
4.8	0.350	3.00	1.918

## Data Availability

Data underlying the results presented in this paper are not publicly available at this time but may be obtained from the authors upon reasonable request.

## Abbreviations

The following abbreviations are used in this manuscript:

RF	ratio frequency
DC	direct current
AC	alternating current
RC	resistance-capacitance
SEM	scanning electron microscope
PIM	passive intermodulation
